# Can Clinical Symptoms and Laboratory Results Predict CT Abnormality? Initial Findings Using Novel Machine Learning Techniques in Children With COVID-19 Infections

**DOI:** 10.3389/fmed.2021.699984

**Published:** 2021-06-14

**Authors:** Huijing Ma, Qinghao Ye, Weiping Ding, Yinghui Jiang, Minhao Wang, Zhangming Niu, Xi Zhou, Yuan Gao, Chengjia Wang, Wade Menpes-Smith, Evandro Fei Fang, Jianbo Shao, Jun Xia, Guang Yang

**Affiliations:** ^1^Imaging Center, Tongji Medical College, Wuhan Children's Hospital (Wuhan Maternal and Child Healthcare Hospital), Huazhong University of Science & Technology, Wuhan, China; ^2^Hangzhou Ocean's Smart Boya Co., Ltd, Hangzhou, China; ^3^Mind Rank Ltd, Hong Kong, China; ^4^School of Information Science and Technology, Nantong University, Nantong, China; ^5^Institute of Biomedical Engineering, University of Oxford, Oxford, United Kingdom; ^6^Department of Radiology, Shenzhen Second People's Hospital, The First Affiliated Hospital of Shenzhen University Health Science Center, Shenzhen, China; ^7^Aladdin Healthcare Technologies Ltd, London, United Kingdom; ^8^British Heart Foundation (BHF) Centre for Cardiovascular Science, University of Edinburgh, Edinburgh, United Kingdom; ^9^Department of Clinical Molecular Biology, University of Oslo, Oslo, Norway; ^10^COVID-19 Specialist Team, Wuhan Children's Hospital, Tongji Medical College, Huazhong University of Science & Technology, Wuhan, China; ^11^Cardiovascular Research Centre, Royal Brompton Hospital, London, United Kingdom; ^12^National Heart and Lung Institute, Imperial College London, London, United Kingdom

**Keywords:** COVID-19, decision trees, machine learning, RT-PCR—polymerase chain reaction with reverse transcription, artificial intelligence, pediatric

## Abstract

The rapid spread of coronavirus 2019 disease (COVID-19) has manifested a global public health crisis, and chest CT has been proven to be a powerful tool for screening, triage, evaluation and prognosis in COVID-19 patients. However, CT is not only costly but also associated with an increased incidence of cancer, in particular for children. This study will question whether clinical symptoms and laboratory results can predict the CT outcomes for the pediatric patients with positive RT-PCR testing results in order to determine the necessity of CT for such a vulnerable group. Clinical data were collected from 244 consecutive pediatric patients (16 years of age and under) treated at Wuhan Children's Hospital with positive RT-PCR testing, and the chest CT were performed within 3 days of clinical data collection, from January 21 to March 8, 2020. This study was approved by the local ethics committee of Wuhan Children's Hospital. Advanced decision tree based machine learning models were developed for the prediction of CT outcomes. Results have shown that age, lymphocyte, neutrophils, ferritin and C-reactive protein are the most related clinical indicators for predicting CT outcomes for pediatric patients with positive RT-PCR testing. Our decision support system has managed to achieve an AUC of 0.84 with 0.82 accuracy and 0.84 sensitivity for predicting CT outcomes. Our model can effectively predict CT outcomes, and our findings have indicated that the use of CT should be reconsidered for pediatric patients, as it may not be indispensable.

## Introduction

Since December 2019, the worldwide spread of coronavirus 2019 disease (COVID-19) has had a significant impact on public health and the global economy. Although most people with COVID-19 manifest mild symptoms, ~20% of patients go through several clinical stages ending in diffuse lung injury, i.e., severe acute respiratory syndrome coronavirus 2 (SARS-CoV-2).

COVID-19 is highly contagious, and severe cases can lead to acute failure of the lungs, multiple organs and ultimately death. The diagnosis of COVID-19 can be confirmed by a laboratory test, i.e., the reverse transcription-polymerase chain reaction (RT-PCR) test; however, the test has high false-negative rates and low sensitivity, which leads to late diagnosis and treatment. Delays in the diagnosis of COVID-19 indicate that patients will amplify the hazard of patient-to-patient COVID-19 transmission within the hospital.

Chest imaging techniques, e.g., chest computed tomography (CT), provides valuable diagnostic and monitoring information that can be used as an important complementary indicator in COVID-19 screening due to high sensitivity (1–4). This is mainly due to most COVID-19 infected patients having chest imaging abnormalities, e.g., bilateral patchy shadows and ground glass opacity (GGO), which are manifested in chest CT scans (5). Meanwhile, subsequent chest CT imaging every 3–5 days are recommended to evaluate the disease progression for fast therapeutic response. Hence, chest CT imaging has become a viable method for early COVID-19 diagnosis and tracking the progression of the disease with high sensitivity. In addition, the WHO Guidelines on Imaging and COVID-19 suggest the diagnostic use of chest imaging for symptomatic patients suspected of having COVID-19 if: (1) RT-PCR testing is not available; (2) RT-PCR testing is available but results are delayed and (3) initial RT-PCR testing is negative but there remains a high clinical suspicion of COVID-19. From a global perspective, imaging techniques are important due to the fact that imaging infrastructures are more advanced in many countries compared to the COVID-19 RT-PCR diagnostic laboratories.

Although chest CT imaging can provide important and complementary diagnostic and prognostic information for COVID-19 patients, some studies believe that the results of CT scans are not highly specific and are not suitable for screening for COVID-19 (6–9). Moreover, multiple chest CT scans have potential carcinogenic effects, which have more prominent risk for vulnerable pediatric patients (10). Besides, for pediatric patients with positive RT-PCR testing results, it is well-known that they can have milder symptoms compared to adults patients (11–13). Despite the fact that chest CT examinations can help us understand the condition of the lungs in pediatric patients (14–16), 35% children with positive RT-PCR testing results can still have negative CT examinations (13, 15), and therefore these patients suffer from unnecessary ionizing radiation (17, 18). Currently, there is no decision support system that can help clinicians to determine whether these pediatric patients with positive RT-PCR testing results need further chest CT examinations.

In this study, we study the relationship between the results of the chest CT examinations and clinical symptoms, laboratory tests and other clinical factors for RT-PCR positive pediatric cases, retrospectively. Using our developed advanced machine learning methods, we establish a systematic decision support system to predict the chest CT results for RT-PCR positive pediatric patients. Our approach will help vulnerable pediatric patients to avoid receiving unnecessary radiation from chest CT scans. At the same time, early predictions of the chest CT results for the pediatric patients using our decision support system can provide better patient classification, clinical decision-making, and more efficient hospital resource allocation.

## Methods

### Datasets

The pediatric patient datasets were collected from Wuhan Children's Hospital. The tabular data contained information for 244 pediatric cases, in which 3 cases had critical COVID-19 symptoms (Table 1). For the feature columns of the tabular data, we collected 32 clinical symptoms for diagnosis (e.g., cough, running nose, sneeze etc.). Following the standard experimental practice, we employed the 5-fold cross-validation for model selection and evaluation. In particular, we split the datasets into five disjoint folds with the same number of samples. Then, we held out each fold for evaluation and the rest 4-folds were used for training our machine learning models. The final result was calculated by averaging over the results of the five experiments. This study was approved by the local Ethics Committee of Wuhan Children's Hospital (Wuhan Maternal and Child Health Care Hospital #WHCH2020005). Written informed parental/guardian consent and child assent (where appropriate) were obtained prior to enrollment in the study.

**Table 1:** Baseline characteristics of children with COVID-19.

	**CT normal** ** (*n* = 102)**	**CT abnormal** ** (*n* = 142)**	**All patients** ** (*n* = 244)**
**Fundamental state**			
Age [Mean (SD)]	7.9 (4.5)	5.6 (4.8)	6.6 (4.8)
Sex (Male/Female)	54/48	85/56	139/104
Contact history	92	126	218
**Symptoms**			
Fever	23	70	93
Cough	23	70	93
Vomit	3	13	16
Diarrhea	2	9	11
Poor spirit	0	7	7
Running nose	4	11	15
**Laboratory examination [median (Range, Q1–Q3)]**			
LDH(U/L)	221 (189–260)	246 (213–326.5)	238 (201–294)
Ferritin (ng/mL)	58.1 (36.8–86.6)	61.6 (40.2–95.3)	58.9 (39.9–90.2)
CK-MB (U/L)	20 (16–32)	24 (18–35)	23 (17–34)
Leukocyte (10^9^/L)	7 (6–8.9)	6.9 (5.4–8.6)	6.9 (5.6–8.7)
Neutrophils (10^9^/L)	3.6 (2.5–4.6)	2.4 (1.7–3.8)	3 (1.9–4.2)
Lymphocyte (10^9^/L)	2.8 (2.3–3.5)	2.9 (2–4.5)	2.9 (2.1–4)
C-Reactive protein (mg/L)	1 (0.8–4)	3 (1–5.9)	1.2 (0.8–5)
Neutrophil lymphocyte ratio (NLR)	1.3 (0.9–1.9)	0.9 (0.5–1.5)	1 (0.6–1.7)

*LDH, Lactic Dehydrogenase; CK-MB, Creatine Kinase-MB*.

### Proposed Methods

It is essential to explore the relationship between the clinical characteristics of children and the COVID-19 RT-PCR testing results. Therefore, an explainable model is required not only to find the implicit relations but can also yield reasonable explanations. Meanwhile, given tabulated data of children who were tested COVID-19 positive or negative, the proposed model should accurately predict the corresponding testing results. We denoted children who were infected by COVID-19 virus (RT-PCR positive) as class 1 and children who were COVID-19 negative (RT-PCR negative) as class 0.

Before building the model, the tabulated data were pre-processed to explore the mean and standard variance of each feature, which provided extra information for mining the relationship. Meanwhile, we also divided the discrete features (e.g., age, leukocyte etc.) into several disjoint intervals which could reduce the complexity of the model.

Besides, feature encoding was also applied due to the fact that some features were not inner correlated. Gender, for instance, was sequentially numbered instead of recorded separately. Therefore, we adopted the one-hot encoding to handle such problems. After pre-processing, we further explored the mutual relationship within the encoded features. We then used the random walk to quantify the strength of the pairwise relations for different features. For example, we found that age had a strong correlation with the contents of the C-reactive protein (CRP).

**Figure 1:**
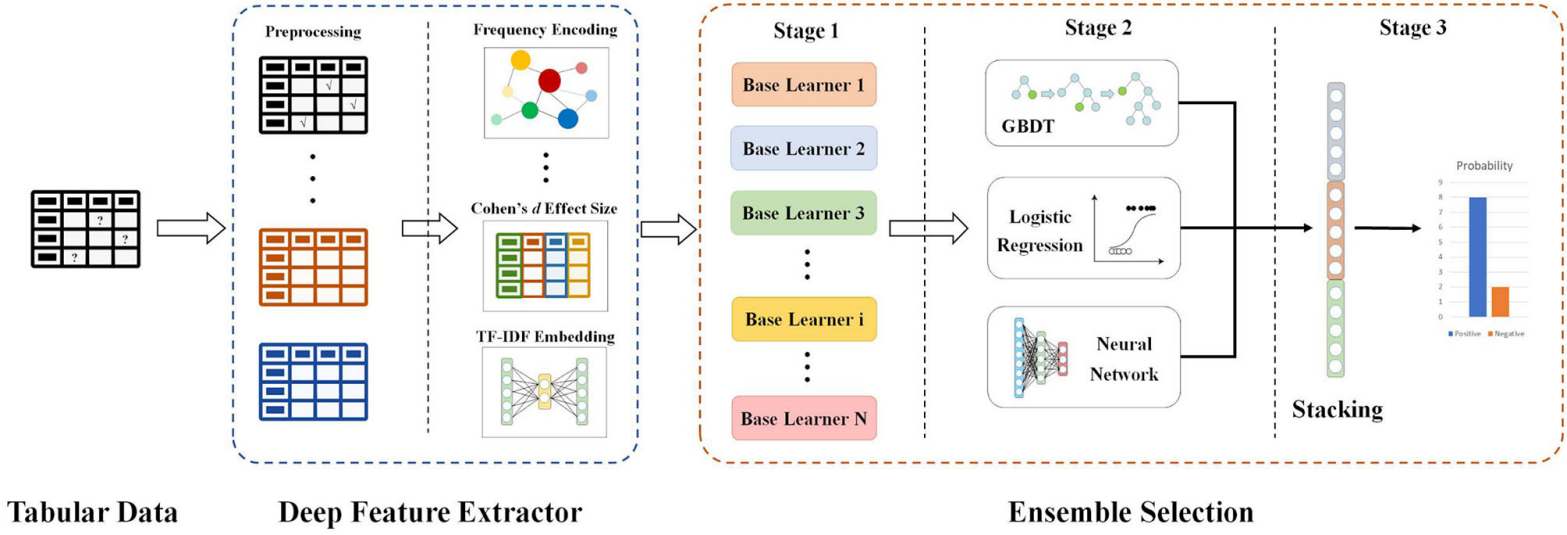
Flow chart and network architecture of our proposed model.

Furthermore, since the contributions of each feature varied, we quantified the importance of features. Features were ranked by measurement generated from algorithms, and we adopted the features with high importance scores to train our model. The ultimate goal of our decision support system is to determine whether CT is required if the RT-PCR test is positive. This is a classification problem with prerequisites; therefore, the interpretability of the model is also very important. Our proposed decision support system (Figure 1) contains the two major modules as follows.

#### An Explainable Feature Extractor Module

##### TF-IDF Embedding

TF-IDF, which stands for Term Frequency–Inverse Document Frequency, is a numerical statistic that can reflect how important a word is to a document in a collection or corpus. A word with higher TF-IDF value is thought to be more important and representative for a document. In this study, for each patient, we extract all the feature values and combines them into a single document. These documents form the whole corpus collection. Then we use TfidfVectorizer from scikit-learn library to find the most important and influential features.

##### Frequency Encoding/Count Encoding

Frequency Encoding/Count Encoding: Both frequency encoding and count encoding are methods to utilize counts of the categories. Since these two methods mainly focus on the frequency and count of each category, they are less affected by the feature values. For example, if two features have similar frequency distribution, we can keep one feature and leave out the other. Although we may miss some information from the discarded features, our model is less likely to overfit as it has less features. In our current study, we develop frequency encoding and apply it to find connections and relationships between features.

##### Target Encoding

Target encoding is a process of replacing a categorical value with the mean of the target variable.

##### Cohen Effect Size

Cohen's *d* is an appropriate effect size for the comparison between two means. To calculate the standardized mean difference *d* between two groups, subtract the mean of one group from the other and divide the result by the standard deviation *s* of the population from which the groups were sampled.

#### An Explainable Classification Module

##### GBDT

Gradient Boosting is a machine learning technique for regression and classification problems, which produces a prediction model in the form of an ensemble of weak prediction models, typically decision trees. It can be fitted to current residuals with gradients of the loss function, in a forward stepwise manner. The GBDT requires no feature normalization and it has an inherently feature selection during the learning process. Besides, it is easy to specify different loss functions for the GBDT.

##### Bayesian Optimization

Bayesian optimization is a sequential design strategy for global optimization for black-box functions that does not assume any functional forms.

Because of the imbalanced nature of the dataset, the traditional training process would lead to unstable performance. In order to tackle unstable training, we divide our dataset into 5-folds and apply the stratified sampling method to ensure each fold's ratio of the positive patients to the negative ones is close to the overall ratio. Furthermore, we adopt the idea of focal loss (19) in our Bayesian optimization process to minimize the influence of the imbalancement.

We used the odds ratio (OR value) to quantify the impact of the individual feature against the output value of our model and the results are reported in Table 2. The OR value in our work referred to the ratio of the exposed patient to the unexposed patient in the positive group divided by the ratio of the exposed patient to the unexposed patient in the negative group. For each feature, if its OR value was >1, it indicated that the factor, which patients were exposed to, was a risk factor that would increase the possibility of being positive. If the OR value was <1, the factor was one protective factor that decreased the chance to be positive. Besides, if the OR value equaled 1 or the confidence interval contained 1, the factor could be considered as irrelevant from a statistical perspective. For example, for feature age, we set the threshold to 7 so the factor is age ≥7. As the OR value was <1 and the confidence interval did not contain 1, so children exposed to this factor, in other words, children who were older than 7 years old were less likely to be positive in CT abnormality than those unexposed, who were under 7 years old.

**Table 2:** Odds ratio for features.

**Feature**	**OR value**	**95% CI**	***p*-value**
Ferritin	10.36	[1.28, 83.69]	0.0196
Lymphocyte	3.11	[1.57, 6.15]	0.0014
C-reactive protein	2.40	[1.42, 4.05]	0.0014
LDH	2.30	[1.12, 4.72]	0.0322
CK-MB	1.67	[0.5, 5.59]	0.5815
Leukocyte	0.47	[0.26, 0.85]	0.0199
Age	0.41	[0.24, 0.69]	0.0011
Neutrophils	0.41	[0.24, 0.7]	0.0016
Neutrophils lymphocyte ratio (NLR)	0.37	[0.15, 0.87]	0.0322

**Figure 2:**
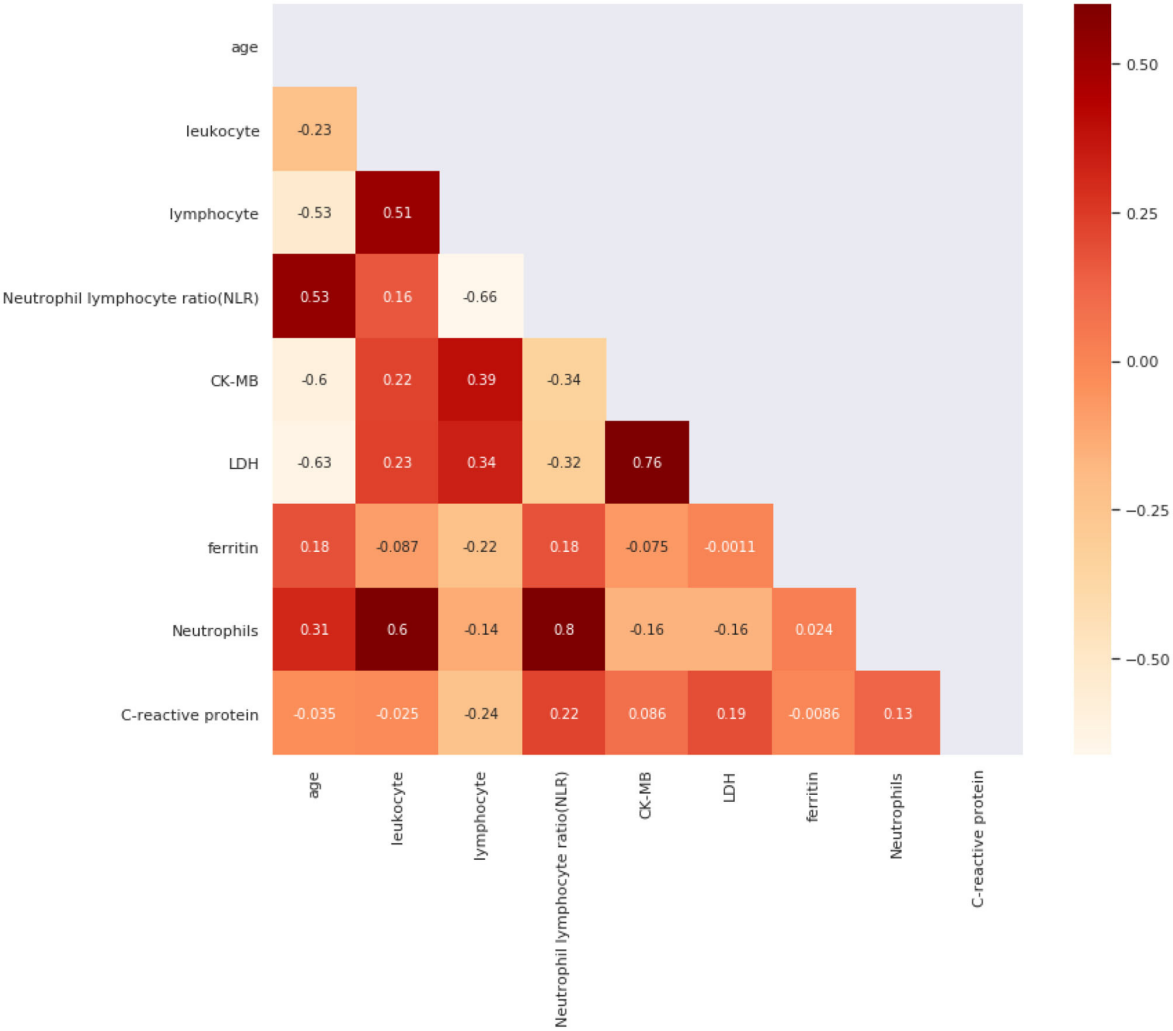
Spearman's Correlation for all features.

We also used Spearman's correlation to find features most related to our target and screened out highly correlated features to minimize input feature numbers. We use a heat map in Figure 2 to present our results. Then we set the threshold value to 0.4 and selected five features out of all the features, which were age, C-reactive protein, Neutrophils, lymphocyte, and ferritin.

However, when we used single-feature models, we could only obtain a relatively fair performance in predicting CT's abnormality. To improve the performance and generalization of our model, the combination of features was necessary. After grouping and aggregating all the patients by their ages and their CT results, we found three significant bounds in ages, which were 4, 7, and 14. We then divided patients into four age groups [0, 4], [4, 7], [7, 14], [14, 16] and calculated the ratio of positive ones to negative ones inside. So, we chose the age as our base feature and combined other features with it.

## Results

As Table 3 shows, compared to conventional and state-of-the-art models, our model has performed significantly better. For instance, our model achieves a higher AUC score of 0.8412, and it is performed better than compared methods by at least 0.8464 for the F1 score. This can be attributed to our effective feature extraction. Compared to our model, TabNet (20), AutoML (21), and DeepFM (22) can only extract the representation of the whole tabular while ignoring representation of the feature itself, which is also important for mining tabular data. Meanwhile, compared with XGBoost (23), we project the feature into higher dimensions with embedding leading to better representation of features. Besides, this leads to an intuitive interpretation, for instance, C-reactive protein may not only indicate the body is healthy or not but can also share a correlation with other indicators (e.g., lymphocyte). Therefore, better feature representation can also lead to better capability of model generalization.

**Table 3:** Comparison of general models.

**Method**	**AUC**	**Accuracy**	**Recall**	**Precision**	**F1 score**
TabNet (20)	0.7891	0.7755	0.7727	0.7391	0.7559
AutoML (21)	0.7453	0.7368	0.7143	0.7895	0.7519
DeepFM (22)	0.6941	0.6818	0.7273	0.6667	0.6970
XGBoost (23)	0.7131	0.7097	0.6429	0.6923	0.6676
Our Model	**0.8412**	**0.8191**	**0.8597**	**0.8389**	**0.8464**

*For all the comparison methods please refer to the opensource implementations at TabNet: https://github.com/dreamquark-ai/tabnet, AutoML: https://github.com/google/automl, DeepFM: https://github.com/ChenglongChen/tensorflow-DeepFM, XGBoost: https://github.com/dmlc/xgboost*.

*Bold values indicate the best performed method*.

To examine the influence of each component and module in our model, we conducted ablation studies, and the results are summarized in Table 4. It can be seen from Table 4 that with the equipment of the encoding procedure, our model can find strong connections between indicators thus has resulted in better performance than the model with GBDT only. Moreover, embedding the features in tabular data and projecting them into higher dimensional space can enrich the representation of features, which improves the model performance on all metrics when Model 1 and Model 3 are compared (Table 4). By incorporating the above two components, our model can achieve a significant improvement by at least 4% on the AUC and 2% on the accuracy.

**Table 4:** Result of all cases where each proposed method can be applied.

**Model**	**GBDT**	**Encoding**	**Embedding**	**AUC**	**Accuracy**	**Recall**	**Precision**	**F1 score**
1	√			0.7081	0.6957	0.7297	0.7105	0.7201
2	√	√		0.7635	0.7581	0.7941	0.7714	0.7828
3	√		√	0.7812	0.7761	0.8158	0.7949	0.8053
4	√	√	√	**0.8412**	**0.8191**	**0.8597**	**0.8389**	**0.8464**

*Bold values indicate the best performed method*.

**Figure 3:**
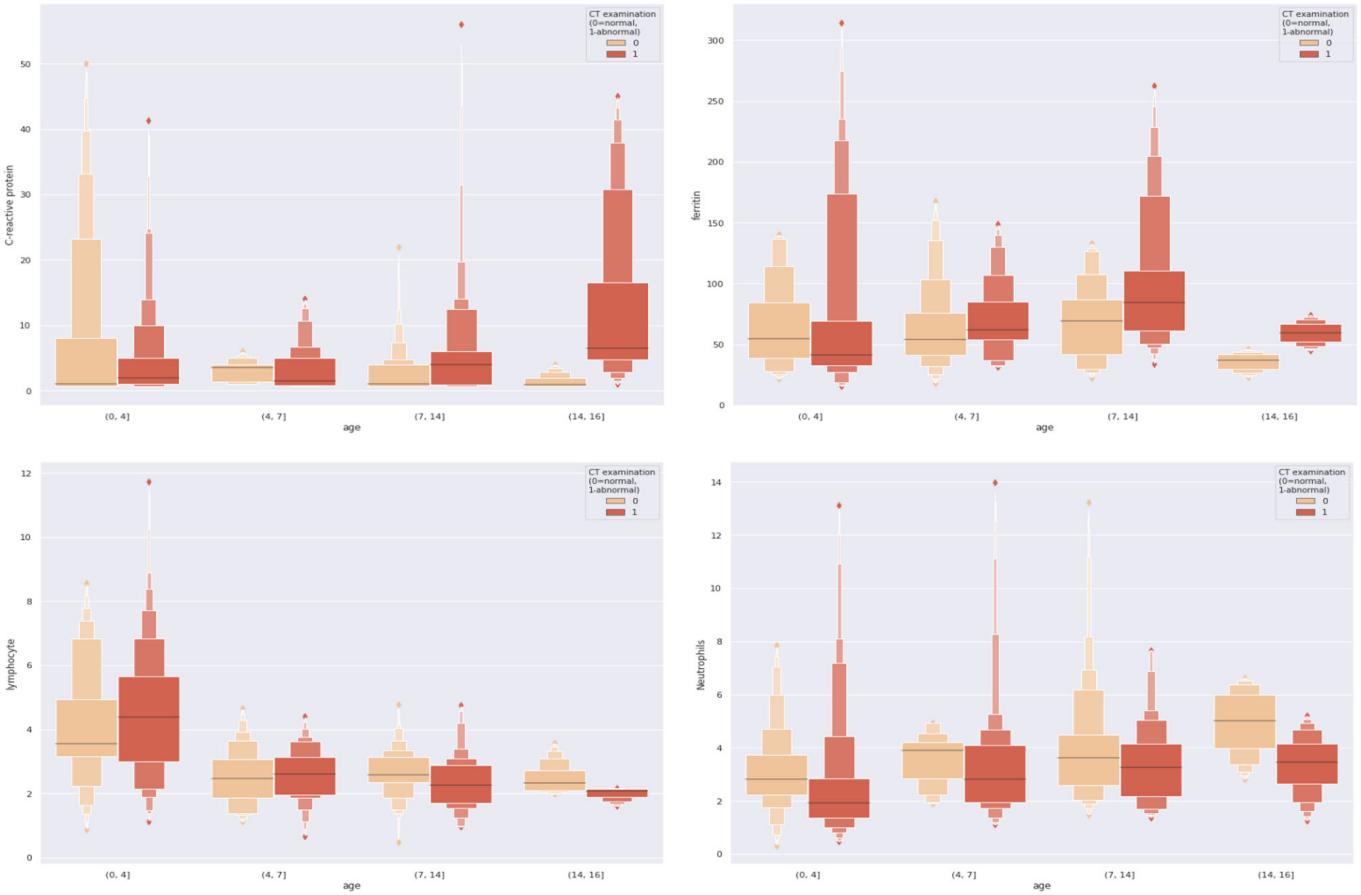
Combinations of different dual features.

To make our work more explicable and understandable, we visualized all the dual combinations. For each patient, we divide patients into different age groups and make them as the x-axis and the combined feature values as the y-axis. The results are demonstrated in Figure 3. We can see significant differences between negative and positive patients when features were combined. For example, with the combination of age and C-reactive protein, we found that for those pediatric patients older than 14 years old, if their C-reactive protein was relatively high, they were more likely to present positive results on CT scans.

**Figure 4:**
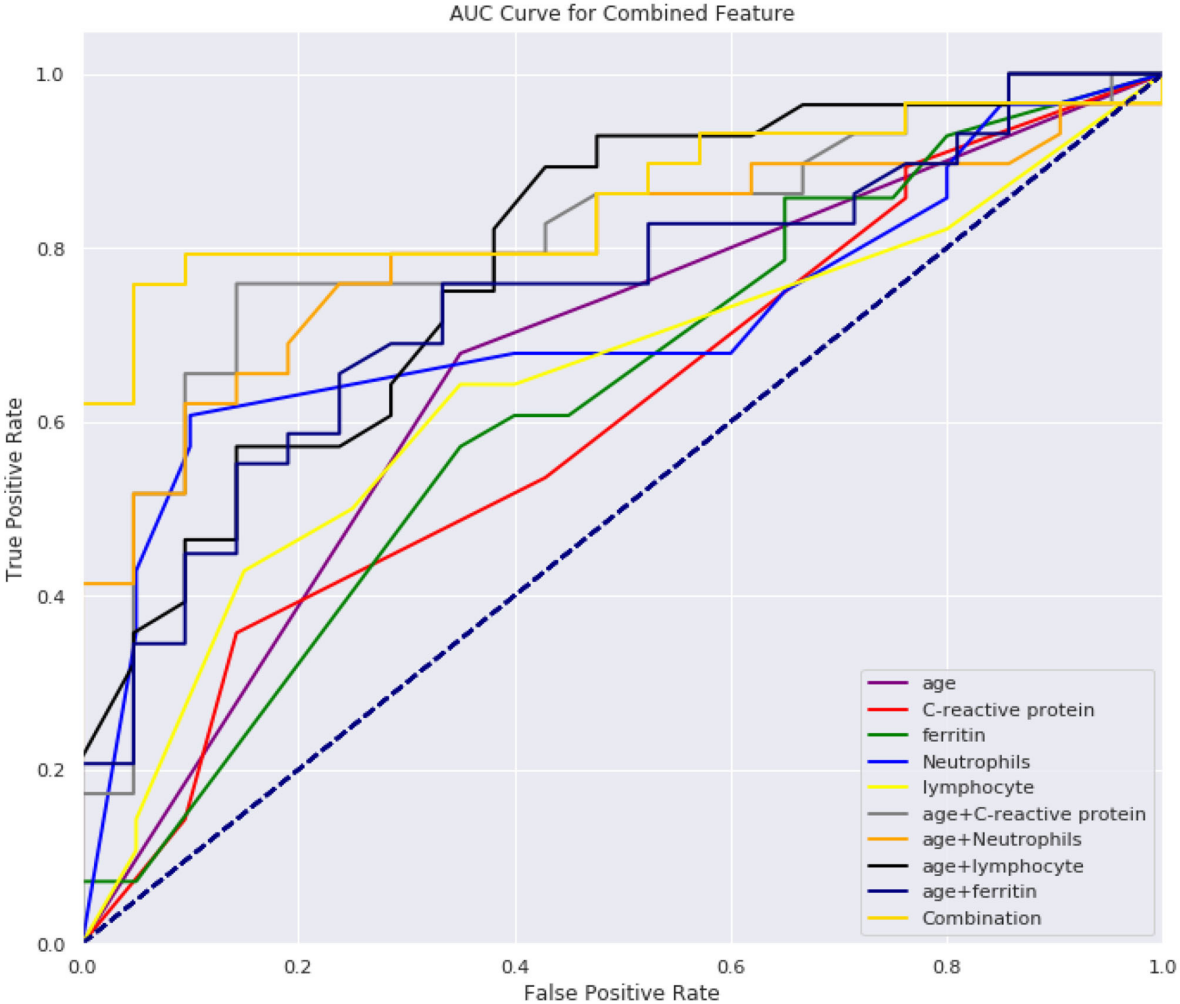
AUC score for all models.

From Tables 5, 6, we can see the performance of our combined-feature models have outperformed single feature models (Figure 4). With all features combined, we managed to get a model achieving AUC score over 0.84 and an accuracy of 0.82. Besides, this model has reached relatively high sensitivity of 0.86, which has indicated that our model is accurate at detecting positive patients, which is quite important for clinical usage.

**Table 5:** Results of single feature models.

**Feature**	**AUC score**	**Accuracy**	**Sensitivity**	**Specificity**	**F1 score**
Age	0.6683 (0.0806)	0.6477 (0.0561)	0.8015 (0.1786)	0.4371 (0.2707)	0.7172 (0.0684)
C-reactive protein	0.5981 (0.0864)	0.6102 (0.0462)	0.7387 (0.2140)	0.4352 (0.3635)	0.6771 (0.0536)
Ferritin	0.5327 (0.1163)	0.6355 (0.0453)	0.8613 (0.1161)	0.3195 (0.2679)	0.7322 (0.0103)
Lymphocyte	0.6194 (0.1047)	0.6355 (0.0659)	0.8500 (0.1225)	0.3424 (0.2990)	0.7302 (0.0266)
Neutrophils	0.6726 (0.0813)	0.6513 (0.0523)	0.8313 (0.1239)	0.4048 (0.2651)	0.7325 (0.0276)

**Table 6:** Results of combined feature models.

**Feature**	**AUC score**	**Accuracy**	**Sensitivity**	**Specificity**	**F1 score**
Age-C-reactive protein	0.8163 (0.1311)	0.7288 (0.0759)	0.8589 (0.0854)	0.5490 (0.2774)	0.7883 (0.0334)
Age-Neutrophils	0.7915 (0.0326)	0.7129 (0.0355)	0.8512 (0.0255)	0.5243 (0.1097)	0.7748 (0.0182)
Age-Ferritin	0.7551 (0.0437)	0.7214 (0.0375)	0.7803 (0.0465)	0.6410 (0.0660)	0.7637 (0.0329)
Age-Lymphocyte	0.7956 (0.0775)	0.7332 (0.0452)	0.7724 (0.0873)	0.6805 (0.0622)	0.7679 (0.0472)
Combination	0.8412 (0.0982)	0.8191 (0.0590)	0.8597 (0.0407)	0.7767 (0.1853)	0.8464 (0.0348)

## Discussion

In this study, we have developed a decision support system which uses five laboratory indicators as inputs and predicts CT scan results of the pediatric patients who have positive RT-PCR testing results.

We found that the combination of five laboratory indicators, i.e., age, C-reactive protein, neutrophils, lymphocyte, and ferritin, can effectively predict whether the CT findings of COVID-19 children are positive or not. The ratio of CT positive to negative is >2 for patients under the age of 4. Between the ages of 4 and 7, the ratio is between 1 and 2; The ratio between 7 and 14 is between 0.7 and 1; >14, the ratio is <0.7. Therefore, we used 4, 7, and 14 years as the cut-off points for predicting CT abnormalities in children, which was proved to be reasonable in our subsequent validation model (Figure 4). We speculate this may be related to the immune system of children. Children under 4 years of age have an immature immune system and weak resistance to the virus (6), which is likely to cause inflammatory changes in the lungs. Therefore, they are more likely to have lung CT abnormalities. Children over the age of 14 have a relatively mature immune system, and at the same time, they have been exposed to places where bacterial or other viral infections are more common, such as nurseries or schools, which allow them to have better-trained immunity, immune fitness and cross-protection (7). It is believed that previous exposure to milder respiratory pathogens can train the immune system of the hosts against the coronavirus (8). Children are less likely to develop severe symptoms of illness as they grow with age, perhaps because the immune system adapts to environmental influences, giving it greater stability (10). Therefore, they are less likely to have lung CT abnormalities.

Neutrophils and lymphocytes, as important components of the innate immune system, have vital functions in the development and recovery of influenza (11). The neutrophil count reflects mostly innate immune cell function, indicating systemic oxidative stress, inflammation, and tissue damage (12). Lymphopenia is very common in patients with influenza virus infection and bacterial infection (13, 14). Ferritin is an acute reactant that is highly expressed in infection and inflammation. Elevated ferritin levels are associated with pro-inflammatory cytokines (15). Ferritin may be a key marker and pathogenic factor in inflammatory pathology, and its signaling pathway is part of innate immune response and regulates lymphocyte function (16).

CRP has been used as a predictor in several previous studies of COVID-19 prediction models (17, 18, 24), and disease progression in MERS, influenza-infected and community-acquired pneumonia patients (25–27). CRP is a marker and indicator of inflammation and plays an important role in host resistance to invasive pathogens and inflammation (28). CRP is elevated in response to inflammation (29) and the level can reflect a persistent state of inflammation which is not affected by factors such as age and gender, detected CRP levels in COVID-19 patients is of great value in assessing the severity of the disease (24, 30, 31). Moreover, CRP was correlated to the acute lung injury in COVID-19 patients (32).

From Figure 3, we can see that the combination of CRP, neutrophils, and ferritin with age is better than these indicators alone. This empirically proves the efficacy of the combination. At the same time, we can also see from Figure 3 that according to the age node we divided before, after combining age with CRP, neutrophils, and ferritin, there are indeed differences among different age groups, which also proves the rationality of our age node division. Finally, we combined age, C-reactive protein, neutrophils, and ferritin, which produced high clinical predictive value. It can be seen that the combined effect is better than the previous pairwise combination (Table 4), and the AUC value can reach to 0.83, which means that through the four indicators of the patient's, we can predict whether the CT appearance of children with COVID-19 is abnormal or not.

In conclusion, in this work, we focus on the explainable features and manage to find some hidden connections between different medical indicators. This is one major advantage of our prediction model compared most current deep learning based black-box models on CT images although different Explainable Artificial Intelligence (XAI) models are currently under development (33–35). The most important contribution of our work is to find five specific indicators out of 32 clinical indicators to predict CT abnormality results. These five indicators, i.e., age, C-reactive protein, Neutrophils, lymphocyte and ferritin, are all easy and quick to obtain under real clinical environment. Thus, pediatric patients with positive RT-PCR testing results may not need to take further CT scans. Besides, we introduced some deep learning methods to the traditional machine learning process. This innovative approach incorporated into our decision support system is a key factor of the success of our model. It is of note that in a recent study (36) it has shown that RT-PCR could yield false negative results at first. To prevent misdiagnosis, the study recommended to isolate patients with normal CT findings but unfavorable RT-PCR outcomes and repeating the RT-PCR. In our current study, we have relied on a single RT-PCR results for model construction and prediction, and we will consider repeating RT-PCR as our future strategy to prevent misdiagnosis and construct more robust gold standard for training the prediction model.

Although our model has outperformed other models for most of the evaluation metrics, there are limitations on the specificity, which means our models may perform less well on predicting negative samples. Moreover, our pediatric patients are all Asian populations, it needs further evaluation to validate if our model could perform well in other human races. These limitations can be eliminated by performing multi-institutional and multi-national studies.

## Data Availability Statement

The datasets presented in this article are not readily available because the paediatric data is under embargo. Requests to access the datasets should be directed to xiajun@email.szu.edu.cn.

## Ethics Statement

This study was approved by the Local Ethics Committee of Wuhan Children's Hospital. Written informed consent to participate in this study was provided by the participants' legal guardian/next of kin.

## Author Contributions

HM, YJ, JX, and GY conceived and designed the study. XZ, YG, CW, WD, ZN, CW, WM-S, EF, and JS contributed to the literature search. HM, JS, and JX contributed to data collection. QY, WD, YJ, MW, JX, and GY contributed to data analysis. HM, QY, MW, XZ, JX, and GY contributed to data interpretation. HM, YJ, MW, XZ, JX, and GY contributed to the tables and figures. HM, YJ, MW, ZN, XZ, JX, and GY contributed to writing of the report. All the authors have read and approved the publication of this work.

## Conflict of Interest

ZN and WM-S are employed by Aladdin Healthcare Technologies Ltd. QY, YJ, and MW are employed by Hangzhou Ocean's Smart Boya Co., Ltd., China and Mind Rank Ltd., China. The remaining authors declare that the research was conducted in the absence of any commercial or financial relationships that could be construed as a potential conflict of interest.
